# Effects of *NM23* transfection of human gastric carcinoma cells in mice

**DOI:** 10.1515/biol-2022-0610

**Published:** 2023-05-23

**Authors:** Na Liang, Chunming Li, Neng Zhang, Qiang Xu, Shengnan Zou, Meng Zhang, Shuyao Si, Li Zeng

**Affiliations:** Department of Histology and Embryology, Zunyi Medical University, Zunyi, Guizhou 563000, China; Department of Pathology, Zunyi Medical University, Zunyi, Guizhou 563000, China; Department of Urology, Affiliated Hospital of Zunyi Medical University, Zunyi, Guizhou 563000, China; Department of Urology, The Second Affiliated Hospital of Zunyi Medical University, Zunyi, Guizhou 563000, China

**Keywords:** gastric carcinoma, NM23, abdominal cancer xenografts, nude mice, metastases

## Abstract

Gastric carcinoma is a frequent malignant tumor worldwide. NM23 plays an important role in pathological processes, including in the occurrence and development of tumors. The purpose of this study is to examine the effect of NM23 transfection of human gastric carcinoma cells (BGC-823) on growth and metastases of BGC-823 abdominal cancer xenografts in nude mice. BGC-823 cells were transfected with an adenovirus vector for *NM23* (NM23-OE), transfected with an empty vector (NC), or were not transfected (Ctrl). Eighteen female BALB/c-nu mice were randomly divided into three groups (six per group) according to the type of BGC-823 cells administered by intraperitoneal injection. After 2 weeks, necropsies of mice were performed, abdominal circumferences were measured, and abdominal cavities were searched by ultrasound. In order to observe the xenografts in nude mice, there were gross macroscopic observations and microscopic observations. In addition, immunohistochemical analysis and western blot of NM23 were also performed. Green fluorescence in the NM23-OE and NC cells indicated successful transfection. The multiplicity of infection is 80%. A comparison of the three groups of mice indicated the NM23-OE group had positive conditions (abdominal circumferences: 81.83 ± 2.40 mm), but the other groups had negative conditions and enlarged abdomens (NC: 90.83 ± 2.32 mm; Ctrl: 92.67 ± 2.07 mm). Ultrasound observations confirmed large tumors in the NC and Ctrl groups, but did not find in the NM23-OE group. There were no obvious ascites in the NM23-OE group, but the cytological examination of ascites exfoliation in NC and Ctrl groups indicated that there were large and deep-stained gastric carcinoma cells. Tumor expression of NM23 was greater in the NM23-OE group than in the NC and Ctrl groups (both *p* < 0.05). In conclusion, transfection of BCG-823 cells with *NM23* rather than an empty vector (NC) or no vector (Ctrl) led to reduced growth and metastases of abdominal cancer xenografts in nude mice.

## 1 Introduction

Gastric carcinoma is a global problem [1], remains the third cause of cancer-related death in worldwide [2], and ranks third in the incidence rate and mortality rate among all cancers in China [3,4]. The risk factors for gastric carcinoma include Helicobacter pylori infection, smoking, overweight and obesity, advanced age, high salt intake, family history of gastric cancer, and so on [5,6,7]. The incidence of gastric carcinoma gradually increases with age [8]. The metastasis of Gastric carcinoma is a complex, multistep pathological process that involves several signaling pathways [9]. Peritoneal dissemination is a common pattern of Gastric carcinoma metastasis and is associated with poor prognosis [10]. Gastric carcinoma cells may adhere to and become implanted in peritoneal mesothelial cells, thus forming metastases that eventually death due to extensive abdominal lesions in the major organs. Extensive research of animal models of human gastric carcinoma has provided insights into the development of gastric carcinoma. The results of research studies have led to the identification and characterization of genes related to gastric carcinoma, the effects of various genetic interventions, and the pathogenic mechanism of different genes. A tremendous amount of results indicated that metastasis of gastric carcinoma is a complex and multi-step process [11].

A number of patients have local invasion and distant metastases when they were identified at first in the hospital. In consideration of the insidious onset of gastric carcinoma, these patients cannot be treated by radical surgery. Even when surgery is combined with chemoradiotherapy for patients with advanced gastric carcinoma, the prognosis remains detrimental [12]. Metastasis is one of the distinguishing features of advanced gastric carcinoma. In particular, gastric carcinoma cells may metastasize remotely through the blood and lymphatic channels or spread from the whole gastric to the abdominal cavity, resulting in rapid deterioration of the patient condition due to the formation of peritoneal implants. The average survival rate of gastric carcinoma patients with peritoneal metastasis is less than 5 months [13], but the 5-year survival rate of patients without peritoneal metastasis is 75% [14].

The nucleoside diphosphate kinase gene *(NM23*) is a cancer suppressor gene. Steeg was the first to discover that NM23 inhibited metastasis by examination of murine melanoma (k-1735) cells [15]. Therefore, measurement of the expression of NM23 in patients with gastric carcinoma may help to predict the risk of metastasis for scholars. In the present study, Zeng et al. constructed a recombinant adenovirus vector (pCMV-NM23-IRES-EGFP) and used it to transfect poorly differentiated human gastric carcinoma cells (BGC-823) [16].

However, the establishment of the human gastric carcinoma animal model can better explore the nature and development of gastric carcinoma and has played a great role in studying the expression of gastric carcinoma-related genes, evaluating the effects of various gene intervention methods and the mechanism of action.

Therefore, this study transplanted these cells into nude mice as a model of human gastric carcinoma and examined the effect of NM23 on the metastasis of gastric abdominal xenograft tumors. The experimental results provide a certain experimental basis for peritoneal metastasis of gastric carcinoma.

## 2 Materials and methods

### 2.1 Cells, vector, and mice

The BGC-823 cells are a line of poorly differentiated human gastric carcinoma cells (HYC3108) and are stored by the laboratory in the Zunyi Medical University. The adenovirus vector (pCMV-NM23-IRES-EGFP) was previously prepared in the laboratory at Zunyi Medical University [16]. The multiplicity of infection (MOI) is 80%.

Experiments were performed on 18 healthy and female BALB/c-nu nude mice, which were 4 weeks old and weighed from 13 to 15 g. The mice were from Chongqing Tengxin Company (certificate number: SCXK [Beijing] 2014-0004) and were raised in a specific pathogen-free animal room of Zunyi Medical University.


**Ethical approval:** The research related to animal use has been complied with all the relevant national regulations and institutional policies for the care and use of animals. All protocols involving the use of experimental animals in this study were approved by the Ethics Committee of Medicine and Science Research Institute of Guizhou Province, and were given a laboratory animals science group reference number: (2012)2-001. The approved programs included the resection, storage, and examination of tumor tissues from nude mice.

### 2.2 Main reagents and equipment

Fetal bovine serum was from Gibco (USA) and NM23 rabbit monoclonal antibody (ab92327) was from Abcam (United Kingdom). MaxVision staining kit for immunohistochemical (IHC) and reagents for western blot were from Solarbio (China). Experiments were performed on an ultraclean workbench (SuZhou Purification Equipment Co., Ltd., China) using an MHG-100B fluorescence microscope from Olympus (Japan) and a gel imaging analysis system from Bio-Rad (USA).

### 2.3 Experimental methods

A recombinant adenovirus vector (pCMV-NM23-IRES-EGFP) was used to transfect BGC-823 cells. The MOI was 80% and the optimal infection volume was 10 µL. They were divided into three groups of BGC-823 cells: the first group is an experimental group (NM23-OE), in which BGC-823 cells were transfected with adenovirus vector (pCMV-NM23-IRES-EGFP); the second group is an empty vector control group (NC), in which BGC-823 cells were transfected with an empty vector (pCMV-MCS-IRES-EGFP); and the third group is an untransfected control group (Ctrl; BGC-823 cells). After 48 h of incubation, the expression of green fluorescent protein (EGFP) in each group was observed by using fluorescence microscopy.

There were six mice in each of the three groups. Each mouse received abdominal cavity injections of 0.2 mL of a cell suspension (1 × 10^7^ cells/mL). Tumor growth was observed, and abdominal circumference was measured every 2 days. Small animal ultrasound (Fujifilm, Japan)was used to detect tumor formation in the abdominal cavity. After 2 weeks, mice were sacrificed by severing their necks and dissected for visual inspection and microscopic observations.

IHC and western blot were used to detect the expression of NM23 in xenograft tumors. The MaxVision two-step method was used for IHC analysis. For IHC, tissue sections were prepared at a thickness of about 4 μm. After antigen retrieval, 50 μl of a goat serum solution was added for blocking (30 min). Then, the primary antibody (NM23 rabbit monoclonal antibody, 1:100) and secondary antibody (goat anti-rabbit IgG antibody, 1:500) were added, and the samples were incubated in a refrigerator at 4°C overnight (about 16–18 h). The DAB solution was added for color development.

For the western blot, total proteins were extracted. The transplanted tumor was placed in the EP tube and immersed it in ice. Then, transplanted tumors were cut by scissors and ground fully in the homogenizer into liquid. Preparation of lysate: put the precooled 1* RIPA buffer into the EP tube, added 10 µl of PMSF (RIPA and PMSF were 100:1) with a pipette per 1 ml of RIPA, mixed well, and split the transplanted tumor in an ice bath. Then, about 500 µl of lysate was needed for every 50 mg of the transplanted tumor. In the process of lysis, the lysed transplanted tumor is transferred into the homogenizer and vibrated once every 5 min, and the transplanted tumor is fully ground to the liquid state, and the whole process was soaked in ice and ground. Then subjected to SDS-PAGE and transferred to a membrane. The membrane was blocked with 5% skimmed milk for 30 min. The primary antibody (NM23 rabbit monoclonal antibody, 1:1,000) and secondary antibody (goat anti-rabbit IgG antibody, 1:500) were added, and the membrane was incubated at room temperature for 1 h. Resolved Strip Grayscale Values were then measured using the Biorad GelDoc XR Imaging System (USA).

### 2.4 Statistical methods

SPSS version 20.0 was used to analyze and process the results. Data were expressed as means ± standard deviations. A one-way analysis of variance was used to compare the three groups, and a *p*-value below 0.05 was considered significant.

## 3 Results

### 3.1 Transfection of cells and establishment of the mouse model

**Figure 1:**
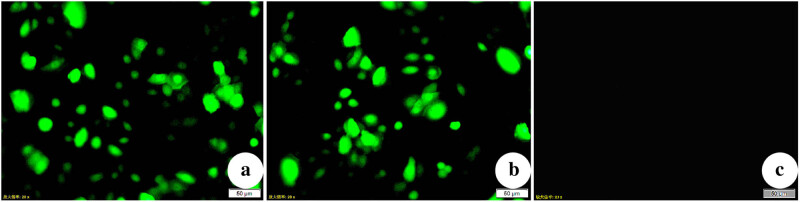
Fluorescence microscopy (×200) of BGC-823 cells after incubation for 48 h in (a) NM23-OE group, (b) NC group, and (c) Ctrl group. Note the presence of fluorescence in (a) and (b), but not in (c).

We incubated the three groups of BGC-832 cells for 48 h with a vector (NM23-OE or NC) or without a vector (Ctrl). The MOI is 80%. Analysis using full-field EGFP fluorescence indicated high expression in the NM23-OE and NC groups, but no expression in the Ctrl group (Figure 1).

In the present study, the apoptosis rate of the NM23-OE group was 12.87%, significantly higher than 1.90% in the NC group and 1.90% in the Ctrl group 1.74% (*p* < 0.05) [16]. Therefore, we then transplanted the different tumor cells into mice. Mice in the NC and Ctrl groups exhibited reduced dietary intake, lethargy, gradual weight gain, and development of a “frog-shaped” belly, suggestive of ascites. In contrast, mice in the NM23-OE group remained normal in appearance. None of the mice died prior to the sacrifice.

**Figure 2:**
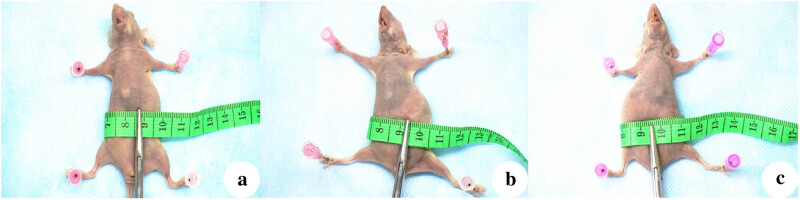
Measure abdominal circumference at 2 weeks after tumor cell transplantation of representative mice in (a) NM23-OE group, (b) NC group, and (c) Ctrl group. Note the larger abdomens in (b) and (c) than in (a).

In research, the basic abdominal circumference was (63.50 ± 1.64) mm in the Ctrl group, (64.33 ± 2.58) mm in the NC group, and (63.50 ± 1.87) mm in the NM23-OE group. The abdominal circumference growth curve showed that the abdominal circumference growth rate in the NM23-OE group was significantly slower than that in Ctrl group and NC group. Two weeks after transplantation, all mice were sacrificed by severing their necks. At that time, the mean abdominal circumference was 81.83 ± 2.40 mm in the NM23-OE group, 90.83 ± 2.32 mm in the NC group, and 92.67 ± 2.07 mm in the Ctrl group. The NM23-OE group had an obviously smaller abdominal circumference than the other two groups (*p* < 0.05, Figure 2).

**Figure 3:**
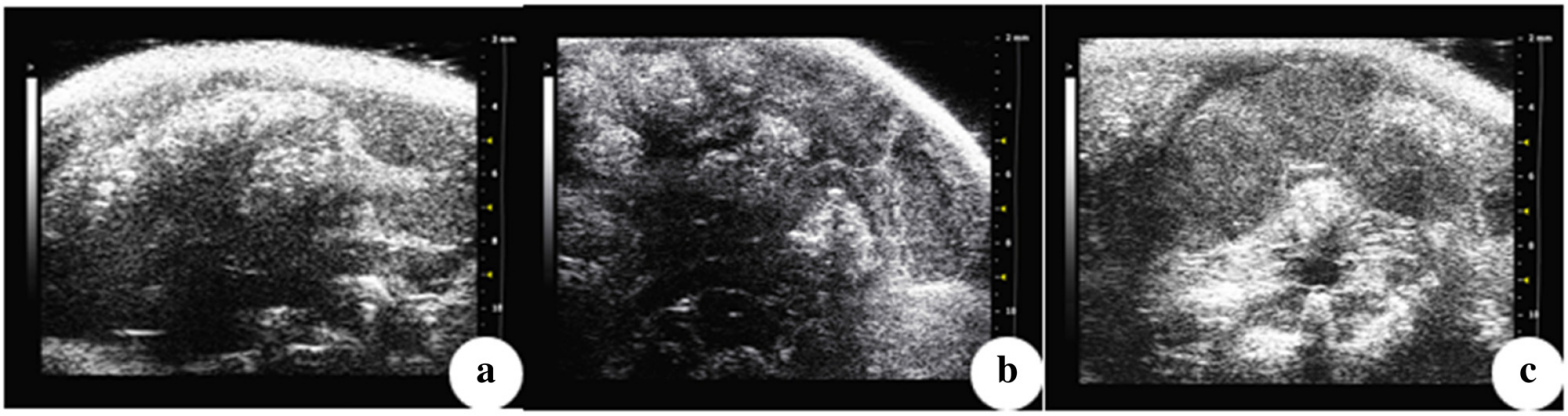
Abdominal ultrasound of representative mice in (a) NM23-OE group, (b) NC group, and (c) Ctrl group. Note the absence of obvious tumors in (a), but tumors metastasis to the intestinal wall in (b) and (c), and tumors metastasis to the peritoneum and hypoechoic nodule in the abdominal cavity (diameter about 0.6 cm) in (c).

The ultrasound results indicated that mice in the NM23-OE group had no large or obvious abdominal tumors (Figure 3). In contrast, ultrasound of mice in the NC and Ctrl groups confirmed large lumps in the abdominal cavity and peritoneal metastases.

### 3.2 Tumor dissection and histology

**Figure 4:**
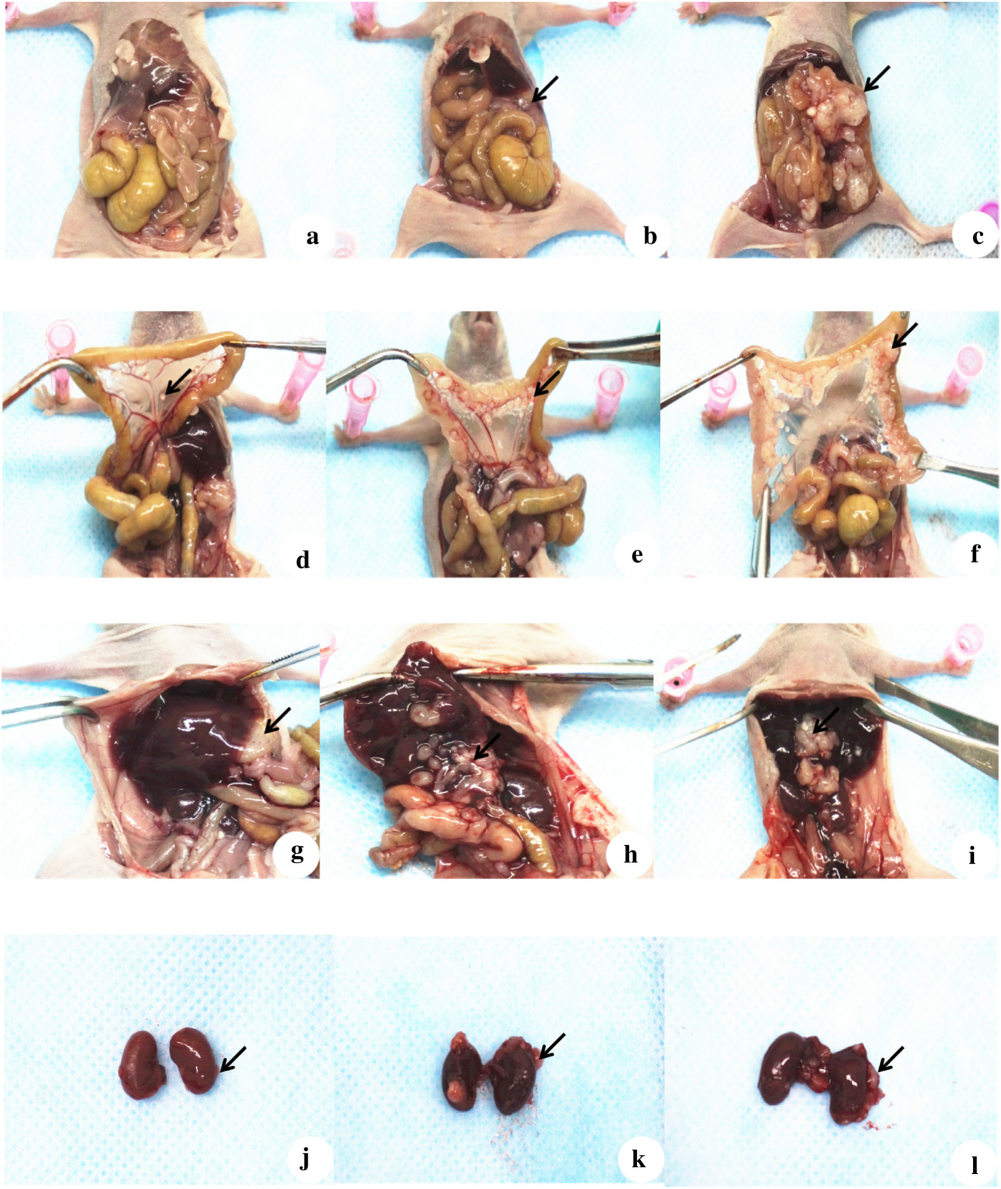
Full abdomen views of representative mice in the NM23-OE group (a), NC group (b), and Ctrl group (c). Note the abdomen in (a) had no obvious tumors, but there was clear evidence of tumors in (b) and (c) (arrows). Mesentery views of representative mice in the NM23-OE group (d), NC group (e), and Ctrl group (f). Note the mesentery in (d) had a diameter of 0.05–0.3 cm, but the mesentery in (e) and (f) had tumors that were round or oval with nodular protrusions, diameters of 0.3–0.7 cm, a gray color, and a hard texture. Livers of representative mice in the NM23-OE group (g), NC group (h), and Ctrl group (i). Note tumor growth in (g) was limited and located in the liver capsule, with a diameter of 0.4–0.5 cm, and no scattered tumor growth in the surrounding liver capsule. The livers in (h) and (i) were large (0.7–0.8 cm in diameter), gray in color, bleeding with necrosis in the center, and with a few scattered and small transplanted tumor growths in the peripheral liver capsule. Kidneys of representative mice in the NM23-OE group (j), NC group (k), and Ctrl group (l). The number of tumors in the kidney capsule was very small in (j), the kidney had a ruddy color, and there was no congestion or enlargement. There were numerous miliary tumors in (k) and (l), and these kidneys were slightly congested and enlarged, and dark in color. The tumors in (k) and (l) were mostly in the upper pole of the kidney and felt firm.

Abdominal dissection of the mice confirmed that the NM23-OE mice had no obvious tumor formations (Figure 4). In contrast, mice in the other groups had a number of xenograft tumors at the site of transplantation. The diameters of these tumors ranged from 0.05 to 0.6 cm in the NM23-OE group. The larger tumors had clear borders, smooth surfaces, easy peeling, no obvious bleeding or necrosis on the cut surface, and these mice had no formation of ascites in the abdominal cavity. Mice in the NC and Ctrl groups also had evidence of tumors in the mesentery, intestinal wall, lower edge of the liver, and kidney capsule.

**Figure 5:**
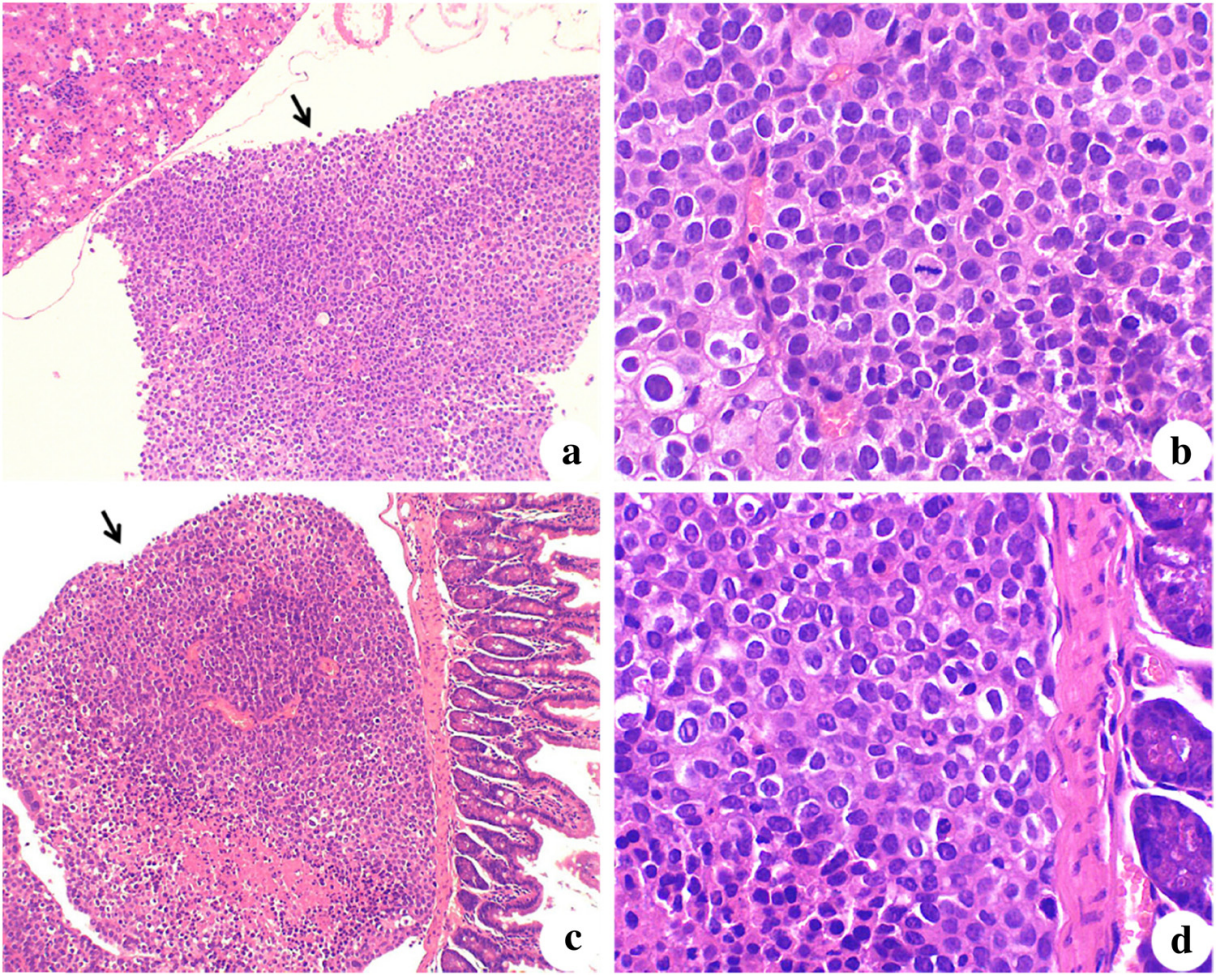
Histological characteristics of tumors from representative mice in (a and b) NM23-OE group and (c and d) NC group (H&E staining). The renal capsule in (a) (×100) indicated that gastric carcinoma cells infiltrated as masses, no involvement of renal parenchyma, and few necrotic tissues. The renal capsule in (b) (×400) indicated a disordered arrangement of gastric carcinoma cells and pathological mitotic figures. The intestinal serosal layer in (c) (×100) indicated the gastric carcinoma cells infiltrated as a mass, without involvement of the intestinal muscle layer and mucosa, and more necrotic tissues. The intestinal serosal layer in (d) (×400) indicated the gastric carcinoma cells had large nuclei and intense staining, with an increased nucleus-to-cytoplasm ratio.

Histological analysis indicated the NM23-OE group had limited gastric carcinoma cell metastasis in the abdominal cavity, with no involvement of renal parenchyma and no evidence of necrosis (Figure 5). In contrast, the renal tissues of NC and Ctrl mice had clear pathological damage, with infiltration of gastric carcinoma cells and necrotic tissues.

**Figure 6:**
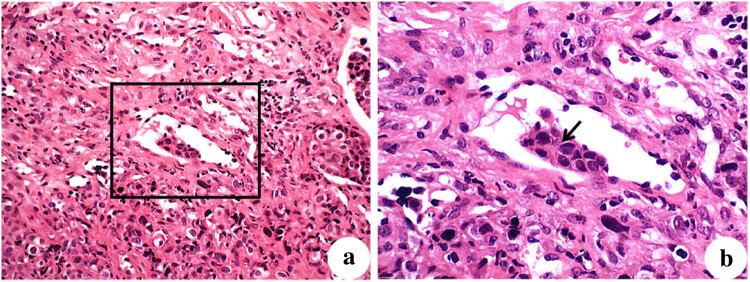
Intravascular tumor thrombus in the gastric muscularis of a representative mouse in the Ctrl group (H&E staining). Gastric carcinoma cells were disorderly and infiltrated the blood vessels of the gastric muscularis in clumps (a, ×200). There was a vascular tumor thrombus in the gastric muscle layer, and gastric carcinoma cells had large nuclei, intense staining, and an increased nucleoplasm ratio (b, ×400).

**Figure 7:**
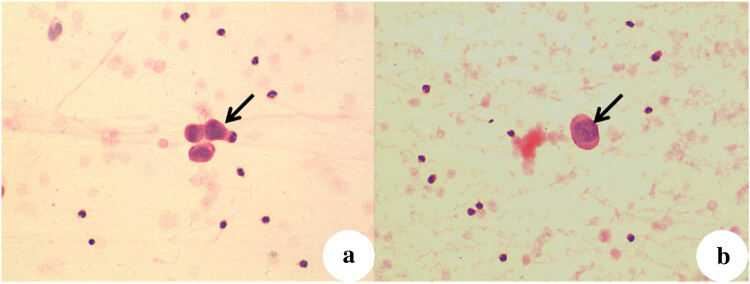
Exfoliated cytology smears (H&E staining, ×400) of ascites in (a) the NC group and (b) the Ctrl group. Note the large cells and large and darkly stained nuclei, mixed with erythrocytes and mesothelial cells.

Further analysis indicated tumor thrombus in the gastric muscularis of the Ctrl group (Figure 6). Multiple serial sections indicated that gastric carcinoma cells infiltrated the gastric muscularis blood vessels. In addition, examination of the ascites of mice in the NC group and the Ctrl group indicated exfoliative cytology and the presence of tumor cells (Figure 7).

### 3.3 NM23 expression

**Figure 8:**
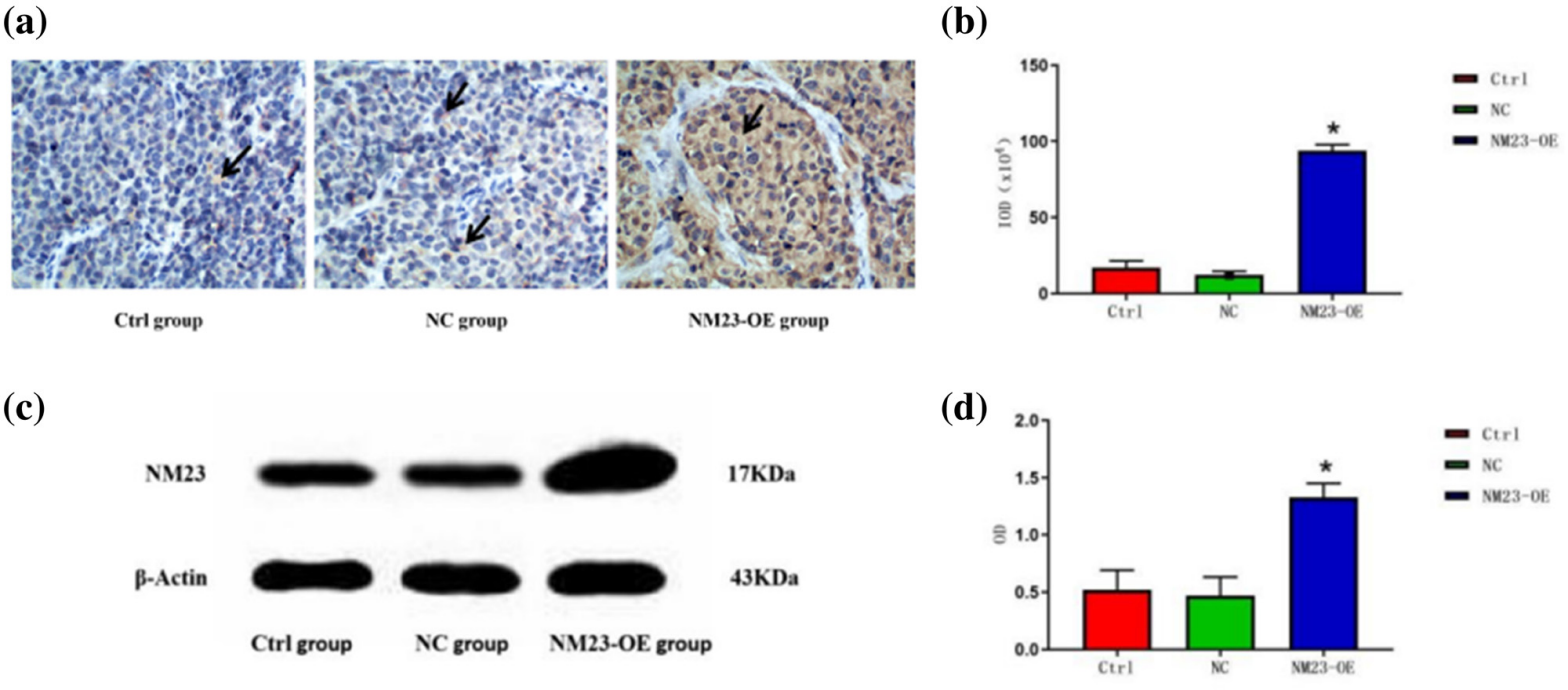
(a and b) IHC analysis and (c and d) western blot of tumor tissues. The expression of NM23 protein in the cytoplasm of cancer cells in the Ctrl group was decreased, NM23 (+). The expression of NM23 protein in the cytoplasm of cancer cells in the NC group was occasionally seen, NM23 (+). NM23-OE group showed sheet or focal positive expression, NM23 (++) (a). An IOD value greater than in the NC and Ctrl groups (*p* < 0.05; (b)). Western blot of tumor tissues confirmed significantly greater expression of NM23 in the NM23-OE group (*p* < 0.05; (c) and (d)).

There was abundant cytoplasmic expression with flaky or focal patterns in NM23-OE group, but scarce expression in NC and Ctrl groups. IHC analysis of the tumors indicated the NM23-OE group had a flaky or focal positive distribution of NM23, and an integrated optical density (IOD) value greater than in the NC and Ctrl groups (*p* < 0.05; Figure 8a and b). Western blot results showed that the average optical density of NM23 protein in the NM23-OE group was increased, indicating that the expression of NM23 protein was higher than that in Ctrl group and NC group, with a statistically significant difference (*p* < 0.05); There was no significant difference in the expression of NM23 protein between the Ctrl group and the NC group (*p* > 0.05; Figure 8c and d).

## 4 Discussion

Tumor progression and metastases are characterized by changes in the expression of multiple genes, and expression of *NM23* is closely related to tumor invasion, metastasis, and altered cells signaling. Gong et al. showed that the cells viabilities of HGC-27 and MKN-45 cells were significantly decreased when the NME2 expression was silenced and the percentage of apoptotic cells was remarkably increased. These data indicated that the NME2 protein took an inhibitory effect on the apoptosis of gastric cancer cells [17]. Recent studies examined the occurrence and progression of gastric carcinoma at the molecular level [18], and gene-targeted therapy has become a research hotspot. There is great potential for the use of gene-targeted therapy as a treatment for patients with gastric carcinoma.

The nude mouse xenograft tumor model is an important experimental model that combines *in vitro* and *in vivo* approaches [19]. Establishing a reasonable and effective animal model is an important prerequisite for research. In this study, we first demonstrated the successful transfection of BGC-823 cells with a recombinant adenovirus vector (PCMV-NM23-IRES-EGFP), based on fluorescence microscopy. We then transplanted these cells into the abdominal cavity of mice, as previously described. After 1 week, mice in NM23-OE group were generally in good condition, had normal activities, and no obvious cachexia. However, mice in the NC and Ctrl groups had reduced food intake, tiredness, abdominal bulges, and weight gain. As the tumor spread, there was no difference in survival of the NC and Ctrl groups. After 2 weeks, the mean abdominal circumference of the NM23-OE group was significantly less than in the NC and Ctrl groups. A small animal ultrasound detector confirmed tumor growth in the abdominal cavities of mice in the NC and Ctrl groups.

Cell adhesion plays an essential role in the two different stages of tumor metastasis. Lacombe. ML showed that NME4 is a metastasis suppressor gene and localized in mitochondria, revealing a prominent role of altered NDPK-D in crucial features of cancer metastasis such as intercellular adhesion, migration, invasion, and EMT [20]; 2 weeks after tumor cells inoculation, intraperitoneal tumors of mice in the NM23-OE group were mainly in the mesentery, lower margin of the liver, and renal capsule, and there were no obvious ascites. The main reason for peritoneal metastasis is that gastric carcinoma cells are free to move within the abdominal cavity, and this can lead to extensive intraperitoneal metastasis. There are many capillaries in the mesentery, and a sufficient blood supply can allow a transplanted tumor to grow rapidly. Therefore, most transplanted tumors were in the mesentery in all three groups, and tumor adhesion was common.

Mice in the NC and Ctrl groups had more organ invasion than mice in the NM23-OE group. The NC and Ctrl mice had widely distributed metastases, whereas the NM23-OE group only had metastases in the mesentery, lower hepatic margin, and renal capsule. We speculate that the mesentery, lower hepatic margin, and surrounding kidney are the sites of early abdominal metastasis of gastric carcinoma. Our IHC and western blot demonstrated higher expression of NM23 in the NM23-OE group than in the other two groups. Thus, these results indicated high expression of NM23 in the intraperitoneal xenografts of nude mice in the NM23-OE group and confirmed that this protein inhibited the metastasis of gastric carcinoma. Ai et al. showed that NM23‐H1 negatively regulated miR‐660‐5p and influenced the proliferation, migration, and invasion. Therefore, NM23‐H1 can inhibit lung cancer bone-specific metastasis [21]. Moreover, predominant melanomas of HPN2 mice grew at a modestly higher rate than those of HP or HPN1 mice; Pamidimukkala. N suggested that the robust metastasis-suppressor activities exhibited by both NME1 and NME2 *in vivo* [22]. The above-mentioned experiments showed that the results were consistent with those of this experimental study. In addition, we will conduct an in-depth study of the gender of different nude mice and the expression of various substantive organs [23].

Clinical research by Zeng et al. confirmed that the expression of NM23 in gastric carcinoma had negatively correlated with histological grade, degree of differentiation, and lymph node metastasis [24]. Wang et al. showed that statistically significant association was found between NM23 expression and the tumor differentiation of patients with gastric carcinoma [25].

Kim and Lee reported that NM23 can be associated with the oxidation of cysteine residues, and affected cancer metastasis [26]. Mátyási et al. announced that NM23-H1 inhibits the proliferation of tumor cells by MAPK signaling and acted against metastatic progression [27]. Sharma et al. emphasized NM23 affected telomeres and telomere-associated functions and inhibited tumor metastasis [28]. Li et al. demonstrated that overexpression of RGS19 which served as GTPase-activating protein (GAP) could activate adenylyl cyclase (AC) and augment intracellular cAMP level, which induces NM23-H1/H2 expression through cAMP/PKA signaling. Moreover, agents such as adrenergic receptor agonists, activator of adenylyl cyclase, and cAMP analogues increase the intracellular cAMP level and the phosphorylation of CREB which significantly increase NM23-H1/H2 expression [29]. However, the identification of the mechanism by which NM23 inhibits metastasis requires further study.
